# Combined measure of salivary alpha-synuclein species as diagnostic biomarker for Parkinson’s disease

**DOI:** 10.1007/s00415-023-11893-x

**Published:** 2023-08-08

**Authors:** Fabrizio Angius, Ignazia Mocci, Tommaso Ercoli, Francesco Loy, Laura Fadda, Maria Francesca Palmas, Giada Cannas, Aldo Manzin, Giovanni Defazio, Anna R. Carta

**Affiliations:** 1 Department of Biomedical Sciences, University of Cagliari, Cagliari, Italy; 2 CNR Institute of Translational Pharmacology, Unit of Cagliari, Cagliari, Italy; 3 Department of Medical Sciences and Public Health, University of Cagliari, Cagliari, Italy; 4 Department of Translational Biomedicine and Neuroscience, Aldo Moro University of Bari, Bari, Italy

**Keywords:** Alpha-synuclein, Biomarkers, Parkinson’s disease, Saliva

## Abstract

Parkinson’s disease (PD) diagnosis is still vulnerable to bias, and a definitive diagnosis often relies on post-mortem neuropathological diagnosis. In this regard, alpha-synuclein (αsyn)-specific in vivo biomarkers remain a critical unmet need, based on its relevance in the neuropathology. Specifically, content changes in αsyn species such as total (tot-αsyn), oligomeric (o-αsyn), and phosphorylated (p-αsyn) within the cerebrospinal fluid (CSF) and peripheral fluids (i.e., blood and saliva) have been proposed as PD biomarkers possibly reflecting the neuropathological outcome. Here, we measured the p-αsyn levels in the saliva from 15 PD patients along with tot-αsyn, o-αsyn and their ratios, and compared the results with those from 23 healthy subjects (HS), matched per age and sex. We also calculated the optimal cutoff values for different αsyn species to provide information about their capability to discriminate PD from HS. We found that p-αsyn was the most abundant alpha-synuclein species in the saliva. While p-αsyn concentration did not differ between PD and HS when adjusted for total salivary proteins, the ratio p-αsyn/tot-αsyn was largely lower in PD patients than in HS. Moreover, the concentration of o-αsyn was increased in the saliva of PD patients, and tot-αsyn did not differ between PD and HS. The ROC curves indicated that no single αsyn form or ratio could provide an accurate diagnosis of PD. On the other hand, the ratio of different items, namely p-αsyn/tot-αsyn and o-αsyn, yielded more satisfactory diagnostic accuracy, suggesting that the combined measure of different species in the saliva may show more promises as a diagnostic means for PD.

**Supplementary Information:**

The online version contains supplementary material available at 10.1007/s00415-023-11893-x.

## Introduction

The diagnosis of Parkinson’s disease (PD) is nowadays based on clinical evaluation and is, therefore, open to bias [25]. The search for biomarkers supporting clinical diagnosis has been intensively pursued, yet with unsatisfactory results. Optimal biomarkers for PD should reflect the underlying pathology and hold high sensitivity and specificity [14]. Detection of alpha-synuclein (αsyn) in the post-mortem brain remains the primary means of reaching a conclusive diagnosis of PD [34]. Physiologically, brain αsyn presents as a soluble monomer that plays a role in intracellular trafficking and synaptic neurotransmitter release [8, 37]. In PD and other synucleinopathies (like dementia with Lewy bodies (LB) and multiple system atrophy), the protein undergoes an aggregation process leading to the final deposition of mature amyloid fibrils and formation of Lewy bodies and neuritis [39]. Such a process involves the formation of several intermediate soluble species, namely oligomers, proto- and pre-fibrils [11, 23, 26, 47]. Moreover, post-translational modifications lead to hyper-phosphorylated forms of αsyn at S129, which are major components of LB [35]. Substantial evidence supports the concept that soluble oligomeric (o-αsyn) and phosphorylated (p-αsyn) species underlie and drive the neurodegenerative process of PD [27, 48]. In the last decade, evidence has pointed at αsyn as a promising in vivo biomarker [9, 30] and several αsyn species have been proposed as PD biomarkers within the cerebrospinal fluid (CSF) and other peripheral fluids such as blood and saliva [5, 22]. In PD CSF, evidence pointed toward a decrease in total αsyn (tot-αsyn) and an increase in p-αsyn and/or o-αsyn [14, 31, 40, 46], changes that possibly reflect the neuropathological scenario. Because of intrusiveness, however, CSF collection is unsuited for use on a routine-basis in PD patients. Therefore, more readily accessible biofluids such as blood and saliva have been evaluated as attractive alternatives. Yet, information from blood markers was often conflicting. Plasma levels of o-αsyn were provided to be more reliable than tot-αsyn [6, 20, 38]; p-αsyn plasma levels were higher in PD patients than healthy controls (HS) but no correlation could be observed with disease progression [20]. Finally, studies on erythrocytes content of αsyn showed more consistent results, with levels of p-αsyn [1, 15, 41] and o-αsyn [29] much higher in PD than in control HS. The αsyn may be measurable in the saliva and in the submandibular gland (SMG), which is the primary source of human salivary volume. Studies reported a decrease in the level of tot-αsyn [4, 17] and significant increase in o-αsyn and o-αsyn/tot-αsyn ratio [36, 44, 45] in the saliva of PD patients in comparison to control subjects. Despite the potential diagnostic value of p-αsyn in the CSF [2, 7], salivary p-αsyn has never been measured as a diagnostic biomarker in PD patients as compared to HS. Of note, p-αsyn has been previously measured in salivary extracellular vesicles from multiple system atrophy-parkinsonism and PD patients, supporting its validity for differential diagnosis [10]. In the present study, we measured the p-αsyn levels in saliva from PD patients along with tot-αsyn, o-αsyn, and their ratios and compared the results with those from HS. We also calculated the optimal cutoff values for different αsyn species to provide information about their capability to discriminate PD from healthy subjects.

## Materials and methods

### Participants

PD patients were recruited among consecutive outpatients attending the Movement Disorders Clinic of the University of Cagliari for diagnosis and follow-up visits. PD was diagnosed by a movement disorder expert in accordance with the Movement Disorder Society Clinical Diagnostic Criteria for PD [33]. Subjects with atypical parkinsonism, dementia, psychiatric conditions interfering with study participation were excluded. Motor severity was assessed by the modified Hoehn and Yahr (HY) scale [24] and the Unified Parkinson’s Disease Rating Scale part III (UPDRS-III) Scale [19]. Moreover, cognitive abilities were assessed with the Montreal Cognitive Assessment (MoCA) [32], and the burden of non-motor symptom manifestations was evaluated by the Non-Motor Symptoms Scale (NMSS) for PD [13]. Data on current medications and disease duration were also collected. Levodopa equivalent daily dose (LEDD) was computed as reported [42]. Controls were healthy subjects (HS) attending neurology outpatient clinics as caregivers or relatives of non-parkinsonian patients; they had no history of PD or any other neurodegenerative disorder. Experimental protocols involving human subjects and sample collection were performed following the guidelines approved by the Local Ethical Committee (approval n. PG/2018/8798) and were subordinate to the acquisition of informed consent from all participants which was then anonymized before use.

### Sample collection and preparation

Saliva samples were collected according to previous studies [44, 45]. In brief, at least 3 ml of saliva were collected from each subject. At the time of collection, subjects had fasted for 2 h, had not smoked in the last 4 h or assumed alcohol in the previous 12 h, and had been examined to have any skin lesion in the oral cavity and no contamination from red blood cells. To obtain an adequate amount of sample, salivation was induced by masticatory stimulus (parafilm chewing for 1 min). Then saliva was collected with a pipette from the sublingual region, poured into a 50 ml sterile test tube containing 50 µl of halt protease and phosphatase inhibitor cocktail (Thermo Scientific, Rockford, lL, USA; Cat #78444). The sample was immediately placed on ice to block the proteolytic activity. Samples were then transferred into new tubes and centrifuged twice for 15 min at 4 °C (2600×*g* and 15,000×*g*, respectively) to remove any fragments or cell debris. Finally, the sample was aliquoted and stored at − 80 °C until analysis.

### ELISA analysis

Before enzyme-linked immunosorbent assays (ELISA), total protein concentration was measured in each sample by BCA Protein Assay kit (Thermo Scientific, Rockford, lL, USA; #23227) to normalize the concentrations of each αsyn form and avoid possible bias due to the variability in salivary protein content. Samples were thawed and centrifuged for 15 min (1000×*g* at 4 °C) and processed for total, oligomeric, and phosphorylated αsyn concentration measurements by ELISA. In line with preliminary experiments (see Supplementary) and according to manufacturer's guidelines, samples were diluted 1:25 for the detection of tot-αsyn and p-αsyn, and 1:2 for the detection of o-αsyn, respectively. Human Synuclein Alpha (SNCa) ELISA Kit (MyBioSource, San Diego, CA, USA; #MBS4502569) was used to reveal total αsyn, Human Alpha Synuclein Oligomer (SNCOa) ELISA kit Sandwich (MyBioSource, San Diego, CA, USA; #MBS730762) for oligomeric αsyn, and Human Phosphorylated Alpha Synuclein (PSNCA) ELISA Kit (MyBioSource, San Diego, CA, USA; #MBS038716) for phosphorylated αsyn. All kits were previously validated in saliva samples in independent preliminary experiments. Each sample from both PD patients and HS was analyzed in three independent experiments in triplicate. The concentration of total, oligomeric, and phosphorylated αsyn was determined by spectrometric measurement at 450 nm using the microplate reader Infinite M200 (Tecan, Männedorf, Switzerland) and calculated by interpolation through the regression analysis using Prism 8 (GraphPad Software, San Diego, CA, USA). Thereafter, for each saliva sample, the measured concentration of specific αsyn species was normalized for 1 mg of total protein.

### Statistical analysis

Statistical analysis was performed using Prism 8 (GraphPad Software, San Diego, CA, USA). Data were expressed as means ± standard errors of the means (SEM) unless otherwise indicated, and analyzed by unpaired Student’s *t* test, Mann–Whitney *U* test, and Chi-square test when appropriate. Significance was set at the 0.05 level. Receiver operating characteristic (ROC) analyses were performed to identify the optimal diagnostic cutoff values for salivary αsyn forms and ratios to discriminate PD patients from controls. To maximize the sensitivity and specificity of the diagnostic tests, the cutoff values were identified by the highest Youden’s Index (sensitivity + specificity–1). The Spearman’s rank correlation coefficient was used to check for correlations between clinical data and the concentrations of αsyn species and their ratios.

## Results

Thirty-eight individuals participated in the study, fifteen PD patients and twenty-three HS. The two groups did not differ for sex (10 men and 5 women vs. 12 men and 11 women, p = 0.5) and age (mean ± SD, 74.7 ± 7.1 vs. 73.9 ± 6.6 years, *p* = 0.7). In PD patients, disease duration (mean ± SD) was 4.1 ± 3.4 years, HY staging (mean ± SD) was 2.1 ± 0.9, *off* UPDRS part III score (mean ± SD) was 27.5 ± 15.9, LEDD (mean ± SD) was 341.7 ± 231.1, MoCA score (mean ± SD) was 21.5 ± 5.3, and NMSS score (mean ± SD) was 57.4 ± 37.4 (Table 1).

**Table 1:** Clinical and demographic features of patients with Parkinson’s disease and healthy subjects

	Parkinson’s disease patients (n.15)	Healthy subjects (n.23)	*p*
Women, *n* (%)	5 (33.3%)	11 (47%)	0.5
Mean age ± SD (y)	74.7 ± 7.1	73.9 ± 6.6	0.7
Mean years of PD duration ± SD (y)	4.1 ± 3.4	NA	NA
HY score	2.1 ± 0.9	NA	NA
UPDRS part III score	27.5 ± 15.9	NA	NA
LEDD, mg	341.7 ± 231.1	NA	NA
MoCA score	21.5 ± 5.3	NA	NA
NMSS score	57.4 ± 37.4	NA	NA

*PD* Parkinson’s disease, *SD* standard deviation, *UPDRS* Unified PD Rating Scale, *LEDD* levodopa equivalent daily dose, *HY* Hoehn and Yahr stage, *MoCA* Montreal Cognitive Assessment, *NMSS* Non-Motor Symptoms Scale, *NA* not available

### Salivary content of salivary proteins and the three αsyn species

**Fig. 1:**
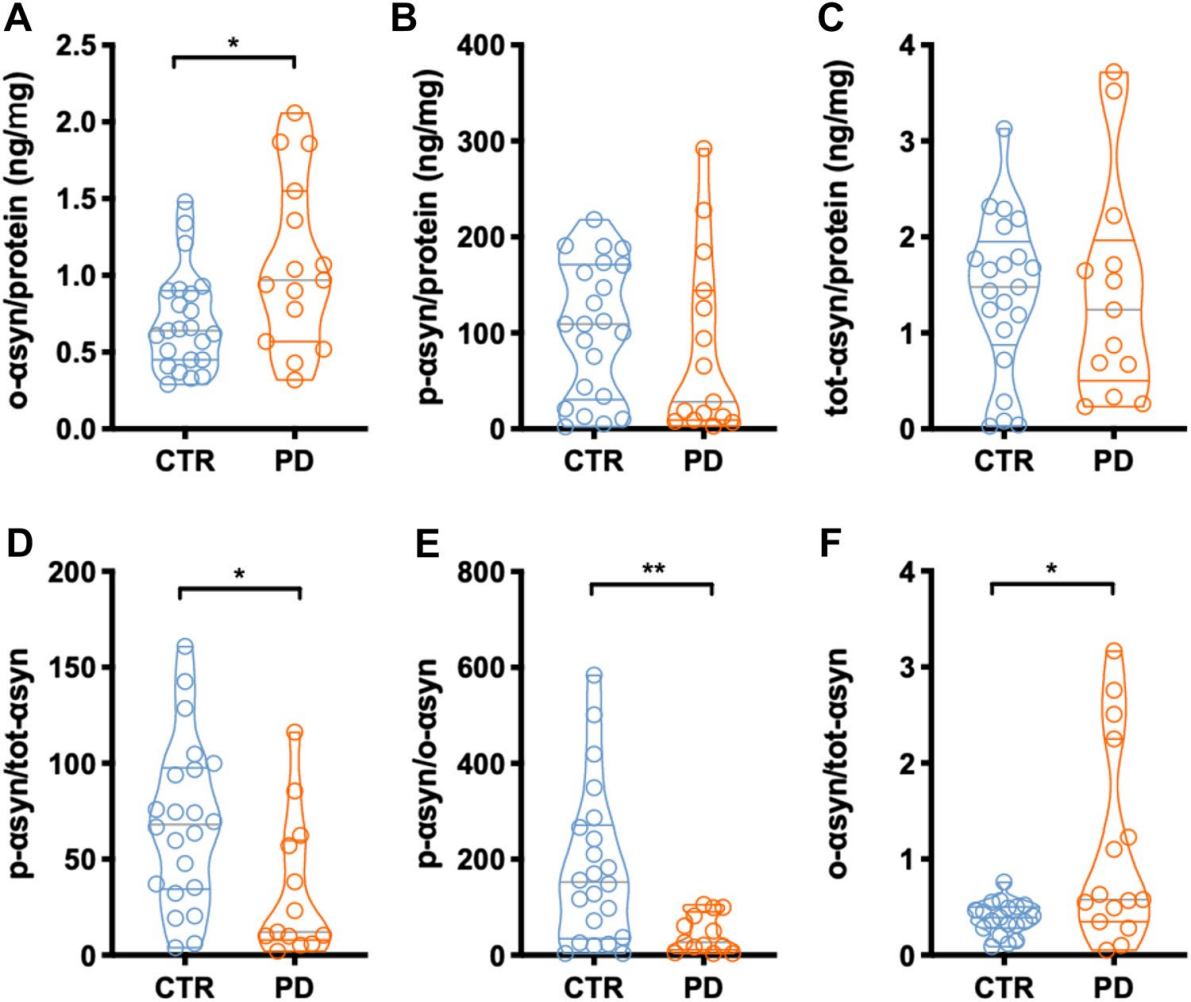
Salivary concentrations of salivary tot-αsyn, o-αsyn, p-αsyn, and the indicated ratios obtained by ELISA in patients with Parkinson’s disease (PD) and healthy subjects (HS). Data are reported as average ± SE of concentrations normalized per mg of total salivary proteins. *p < 0.05 and **p < 0.01

BCA analysis revealed a significantly lower amount of total proteins in PD patients as compared to HS (2044 ± 227.5 vs. 2963 ± 236.3 μg/ml, *p* = 0.02). Although o-αsyn and p-αsyn were measurable in the saliva samples of both PD and HS, levels of o-αsyn were in the range of 0.5–2 ng whereas p-αsyn was more abundant, being in the range of 100–200 ng. Furthermore, o-αsyn content was significantly higher in PD patients as compared to HS (Fig. 1a). Instead, the p-αsyn concentration was comparable in PD patients and HS (Fig. 1b) and, similarly, tot-αsyn content did not differ significantly between PD patients and HS (Fig. 1c). When we interrelated these measures by calculating their ratio (Fig. 1d–f), the p-αsyn/tot-αsyn ratio (Fig. 1d) as well as the p-αsyn/o-αsyn ratio (Fig. 1e) were significantly lower in the saliva from PD patients than HS. By contrast, the o-αsyn/tot-αsyn ratio was significantly higher in PD vs. HS (Fig. 1f). Moreover, we further investigated possible correlations between αsyn values and clinical parameters in PD by Spearman’s correlation analysis and among those we found that tot-αsyn was negatively correlated with UPDRS-III (*r* = − 0.7785, *p* = 0.0025; Fig S2) and that the p-αsyn showed a negative correlation with NMSS (*r* = − 0.6154, *p* = 0.0165; Fig S2). Finally, any correlations were found between the o-αsyn or αsyn ratios and the PD patients’ clinical data.

### Sensitivity and specificity evaluation

**Fig. 2:**
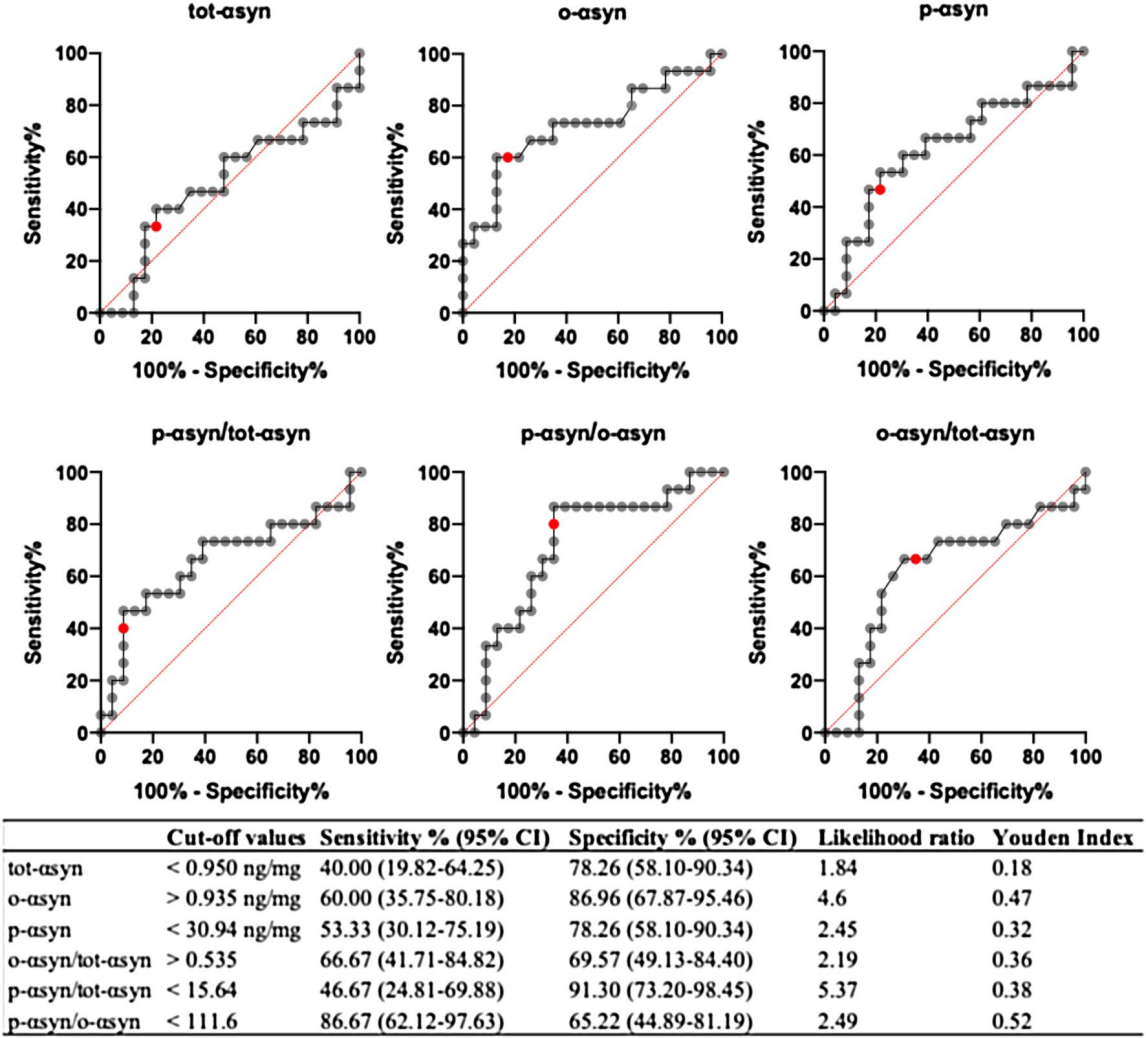
Receiver operating characteristic (ROC) analysis showing sensitivity and specificity of salivary total, oligomeric, and phosphorylated αsyn concentration as well as phosphorylated/total, phosphorylated/oligomeric and oligomeric/total αsyn ratio values used to discriminate PD patients from HS. Likelihood ratio and Youden Indexes were performed to identify the optimal cutoff values (red spots in the graphs) to differentiate PD from HS. CI: 95% confidence interval

On ROC analysis, the highest YIs—identifying the optimal diagnostic cutoff values for salivary αsyn forms—were 0.47 (cutoff value, 0.935 ng/mg) for o-αsyn, 0.36 (cutoff value, 0.535) for o-αsyn/tot-αsyn ratio, 0.38 (cutoff value, 15.64) for p-αsyn/tot-αsyn ratio, and 0.52 (cutoff value, 111.6) for p-αsyn/o-αsyn ratio (Fig. 2). By applying these cutoff values, no individual item reached a satisfactory combination of sensitivity/specificity; however, the greatest sensitivity (87%) was obtained by the p-αsyn/o-αsyn ratio while the best specificity (91%) was reached by the p-αsyn/tot-αsyn ratio (Fig. 2). We, therefore, assessed whether a combination of items could improve diagnostic accuracy (Table 2). The best sensitivity and specificity were achieved by the combination of p-αsyn/tot-αsyn ratio and o-αsyn that yielded 80% sensitivity and 78% specificity.

**Table 2:** Sensitivity and specificity of individual α synuclein parameters and their combination

Item or combination of items (cut off value indicating PD)	Sensitivity (n. identified PD/total n. PD patients)	Specificity (n. identified control subjects/total n. control subjects)
O (> 0.935)	60% (9/15)	87% (20/23)
P (< 30.94)	53% (8/15)	78% (18/23)
P/O (< 111.6)	87% (13/15)	65% (15/23)
P/T (< 15.64)	47% (7/15)	91% (21/23)
O/T (0.535)	67% (10/15)	69% (16/23)
P/O (< 111.6) + P/T (< 15.64)	87% (13/15)	65% (15/23)
P/O (< 111.6) + P (< 30.94)	87% (13/15)	65% (15/23)
P/O (< 111.6) + O/T (> 0.535)	93% (14/15)	56% (13/23)
P/O (< 111.6) + O (> 0.935)	87% (13/15)	65% (15/23)
P/T (< 15.64) + P/O (< 111.6)	87% (13/15)	65% (15/23)
P/T (< 15.64) + P (< 30.94)	40% (9/15)	78% (18/23)
P/T (< 15.64) + O/T (> 0.535)	93% (14/15)	65% (14/23)
P/T (< 15.64) + O (> 0.935)	**80% (12/15)**	**78% (18/23)**
P (< 30.94) + P/T (< 15.64)	40% (9/15)	78% (18/23)
P (< 30.94) + P/O (< 111.6)	87% (13/15)	65% (15/23)
P (< 30.94) + O/T (> 0.535)	87% (13/15)	61% (14/23)
P (< 30.94) + O (> 0.935)	87% (13/15)	69% (16/23)
O (> 0.935) + P	87% (13/15)	69% (16/23)
O (> 0.935) + P/T	**80% (12/15)**	**78% (18/23)**
O (> 0.935) + P/O	87% (13/15)	61% (14/23)
O (> 0.935) + O/T	80% (12/15)	65% (15/23)
O/T (0.535) + P	87% (13/15)	61% (14/23)
O/T (0.535) + P/T	93% (14/15)	61% (14/23)
O/T (0.535) + O	80% (12/15)	65% (15/23)
O/T (0.535) + P/O	93% (14/15)	56% (13/23)

The parameters combination achieving the best sensitivity and specificity are marked in bold

## Discussion

In the present study, we measured the content of total and oligomeric species of αsyn and the content of phosphorylated αsyn in the saliva of PD patients and HS. Concentration of oligomeric and phosphorylated αsyn was either adjusted for total salivary proteins or for total αsyn content. We found that p-αsyn was the most abundant αsyn species in the salivary fluid but its concentration did not differ between the two populations when adjusted for total salivary proteins. However, the ratio p-αsyn/tot-αsyn was largely lower in PD patients than in HS. Moreover, the concentration of o-αsyn was increased in the saliva of PD patients, while tot-αsyn did not differ between PD and HS. The ROC curves indicated that no single αsyn form or ratio could provide an accurate diagnosis of PD. On the other hand, the combination of different items, namely p-αsyn/tot-αsyn and o-αsyn, yielded more satisfactory diagnostic accuracy.

### Measure of salivary αsyn species in PD and HS

The results of the salivary assay were consistent with previous studies showing higher o-αsyn content in PD saliva as compared to HS [44, 45]. Moreover, we did not detect any difference in the tot-αsyn content between the two groups, while in previous studies, the salivary content of tot-αsyn in PD was decreased or unvaried [12, 44]. To this regard, it is noteworthy that the present study was conducted in a relatively small sample of subjects, falling in a narrower range of clinical features such as PD duration, HY and UPDRS-III score in comparison with previous studies. Our study also provided novel information on salivary p-αsyn, showing that this species was highly present in the saliva, and its concentration over tot-αsyn was significantly lower in PD patients than HS. The p-αsyn has been previously suggested as a potential biomarker for PD when measured in the CSF, plasma, and SMG of PD patients [21]. In the SMG, p-αsyn has been previously described both in PD patients and elderly HS. Interestingly, SMG p-αsyn was generally described in the perikaryal cytoplasm in both PD and HS, while it was additionally located in nerve fibers of PD patients [2, 3, 7]. We can speculate that salivary p-αsyn is released from nerve fibers, and that salivary content may reflect the nerve fibers content. In this regard, more studies are warranted to investigate any correlation of salivary p-αsyn with clinical parameters in PD. While measurement of p-αsyn in the SMG biopsy has been suggested as a potential diagnostic biomarker, salivary measure is a repeatable non-invasive assay which also overcome caveats related to asymmetry in SMG described in previous studies [3]. In the present study, the salivary o-αsyn and p-αsyn displayed an opposite trend in PD patients, being respectively increased and decreased with respect to HS. These toxic species are both involved in PD neuropathological processes. Phosphorylation at S129 is a post-translational modification of αsyn. In turn, hyper-phosphorylation in the CNS promotes the aggregation process and the formation of soluble oligomers, ending in the final aggregation of insoluble fibrils. Hence, LBs are enriched in hyper-phosphorylated protein. Although the present study was conducted in a limited population size, we investigated possible associations between salivary levels of αsyn species and clinical features. We found that tot-αsyn negatively correlated with the UPDRS-III, indicating that tot-αsyn salivary concentration decreased in relation with the severity of symptoms. Moreover, p-αsyn was negatively correlated with NMSS, indicating a decrease in relation with the severity of non-motor symptoms. It is worth to notice that the p-αsyn is highly prone to aggregate leading to more rapid oligomerization than monomeric αsyn. Accordingly, we found o-αsyn to be significantly increased in PD vs. HS.

### Diagnostic accuracy of salivary assays

With regard to diagnostic accuracy of αsyn species and their ratio, no study item alone allowed an accurate diagnosis of PD. Nevertheless, combining p-αsyn/tot-αsyn ratio and o-αsyn yielded 80% sensitivity and 78% specificity. This means that the combination of the two items correctly diagnosed PD in 8/10 patients who have the condition, and correctly identified as HS about 8/10 subjects. The combination of p-αsyn/tot-αsyn ratio and o-αsyn may, thus, be considered as a promising diagnostic tool, particularly when we compared our findings to those reached by other methodological approaches that are summarized in Table 3. CSF tot-αsyn distinguished PD and controls with 75% sensitivity and 65% specificity, while CSF o-αsyn yielded 71% sensitivity and 64% specificity [18]. Better results were obtained by skin biopsy. In fact, a recent meta-analysis showed that pooled sensitivity and specificity of anti-p-αsyn antibody in the skin of PD and HS were 76% (0.69–0.82) and 100% (0.98–1.00), respectively [43]. However, skin biopsy has several methodological problems, including the biopsy site choice, section thickness, antibody selection, and the requirement of skilled personnel for specimen collection and processing. Finally, skin biopsy may not be well-tolerated by some patients. With regard to imaging approaches, the diagnostic accuracy of DatScan imaging, one of the most commonly used techniques in clinical practice, is similar to the clinical diagnostic accuracy in terms of sensitivity (84–98%) and specificity (67%) [16]. Transcranial sonography (TCS) is a non-invasive neuroimaging technique that can visualize the substantia nigra and may detect changes related to PD. A meta-analysis of 31 studies containing 4,386 participants reported a pooled sensitivity of 0.83 (95% CI 0.81–0.85) and a pooled specificity of 0.87 (95% CI 0.85–0.88) [28]. Despite these encouraging results, the routine use of TCS as a diagnostic tool needs caution because its accuracy may vary according to the expertise of the operator, the equipment used, and the selection of study subjects.

**Table 3:** Suggested biomarkers for Parkinson’s disease

References	Methodological approach	Control sample	Sensitivity	Specificity	Limitation of the technique
Eusebi et al. [18]	CSF tot-αsyn	HS and patients with other neurological disorders (including parkinsonism)	75%	65%	Invasive procedure and risk of complications; limited use in certain medical conditions
	CSF o-αsyn		71%	64%	
Tsukita et al. [43]	Skin biopsy (anti-p-αsyn antibody)	HS and patients with other neurological disorders (including parkinsonism)	76%	100%	Issues in the choice biopsy site, section thickness, and antibody selection. Requirement of skilled personnel for sample collection and processing. Not well-tolerated by some patients
de la Fuente-Fernández [16]	DatScan	HS and patients with parkinsonism	84–98%	67%	Mildly invasive procedure; limited availability; interpretation variability
Li et al. [28]	Transcranial sonography	HS	83%	87%	High variability depending on the operator expertise, the equipment used, and the selection of study subjects

### Our study has limitations and strengths

This study was performed in a small sample of PD patients that, in addition, was not population based. Thus, we could not rule out a selection bias. This limitation notwithstanding, the consecutive recruitment of patients during the study period, as well as the diagnosis made by experts in movement disorders yielded a clinical series resembling the general population of PD in an early stage of disease. We excluded first-diagnosed patients before chronic dopaminergic drug administration because the benefit by dopaminergic drugs would support PD diagnosis. Bias caused by the examiners being unblinded to the case/control status was unlikely because they were unaware of the results of αsyn assay. Our control group included only HS while other diagnostic methodologies [16, 18, 43] also focused on patients with other neurological diseases. In this regard, it is worth noting that clinical examination can often exclude non-parkinsonian neurodegenerative diseases, whereas most diagnostic difficulties arise from parkinsonian-like syndromes that may be included within the synucleinopathies (dementia with LB and multiple system atrophy) or not (progressive supranuclear palsy and corticobasal degeneration). Future confirmatory studies should, therefore, assess salivary synuclein parameters also in these conditions. Finally, the mere analytical methods may suffer from limitations due to the nature of the sample that may interfere with the analyte detection—known as matrix effect—easily overcome by appropriate and accurate preliminary validation steps (see Supplementary).

## Conclusion

Despite the foregoing limitations, our study confirms that different αsyn species may be detectable in the saliva from PD patients and provides novel information on salivary p-αsyn in PD. The exploratory analysis about the use of αsyn species and ratios from the salivary fluid as diagnostic biomarker in PD suggests that the combined measure of different αsyn species in the saliva may show more promise than single αsyn species or ratio. Our results should prompt further research efforts in larger samples of patients with PD and other synucleinopathies and controls (also including patients with other neurological diseases) to evaluate the usefulness of salivary p-αsyn/tot-αsyn and o-αsyn as preclinical/clinical diagnostic biomarkers.

### Supplementary Information

Below is the link to the electronic supplementary material.

Supplementary file1 (DOCX 313 KB)

## Data Availability

Data of the present study will be made available upon request.
