# Supplementary material for: Combined measure of salivary alpha-synuclein species as diagnostic biomarker for Parkinson’s disease
Source: J Neurol. 2023 Aug 8;270(11):5613–21. doi: 10.1007/s00415-023-11893-x (PMC10576686; doi:10.1007/s00415-023-11893-x)
Supplement: Supplementary file 1 — Supplementary file1 (DOCX 313 KB) [file 415_2023_11893_MOESM1_ESM.docx]

**Combined measure of salivary alpha-synuclein species as diagnostic biomarker for Parkinson’s disease**

Fabrizio Angius^1^, Ignazia Mocci^2^*, Tommaso Ercoli^3^*, Francesco Loy^1^, Laura Fadda^3^, Maria Francesca Palmas^1^, Giada Cannas^1^, Aldo Manzin^1^, Giovanni Defazio^3,4^@, Anna R. Carta^1^.

^1^Department of Biomedical Sciences, University of Cagliari, Cagliari, Italy

^2^CNR Institute of Translational Pharmacology, Unit of Cagliari, Cagliari, Italy

^3^Department of Medical Sciences and Public Health, University of Cagliari, Cagliari, Italy;

^4^Department of Translational biomedicine and neuroscience, Aldo Moro University of Bari, Bari, Italy

* authors equally contributed

Corresponding author:

Giovanni Defazio ([giovanni.defazio@uniba.it](mailto:giovanni.defazio@uniba.it))

**Supplementary Information**

**ELISA assay validation**

To validate and assess the accuracy of the assay we performed a spike-and-recovery experiment in order to determine whether analyte detection might be affected by the interference, known as matrix effect, due to the particular sample type. For p-αsyn, a different amount of standard analyte was added into the sample (spike) and measured by comparison to an identical spike in the sample diluent used as standard curve. The standard curve showed a high goodness of fit (Rsq = 0.9985; Sy.x = 0.0273) and allowed the interpolation of data. Based on the spike-and-recovery results (Table S1 and Fig. S1), we chose the 1:25 dilution as appropriate to minimize the interference in the analyte detection. In fact, the spike-and-recovery experiment revealed that when a sample is challenged with lower to higher standard concentration (3.12 to 25 ng/ml) the 1:2.5 dilution results in highly inconsistent data (Fig. S1B), whereas dilution between 1:10 and 1:50 was more consistent. Moreover, the recovery results using a 1:10 and 1:50 dilutions factor results in an average recovery percentage of 114.39 and 106.94, respectively. Accordingly, the use of 1:25 dilution factor grants a recovery rate in line with the widely used values reported in validation protocol for ELISA assay. The same procedure was applied for total and oligomeric forms of αsyn. In addition, in order to test the assay mimicking a real sample, we pooled the samples from PD patients and processed toward the quantification of p-αsyn. Figure S1C depicts the curve obtained by several dilutions and reveals consistent linearity-of-dilution results between 1:10 to 1:100. Moreover, we calculated the real concentration by multiplying the measured concentration to the relative dilution factor and, as reported in panel D, the higher the dilution factor the less accurate the concentration is, as indicated by progressively wider error bars (Fig. S1D).

**Table S1. Spike-and-recovery results for phosphorylated alpha-synuclein (p-αsyn) in human saliva samples.** Dilutions were made in a previously defined diluent and observed concentration values were measured by interpolation to the assay standard curve.

| **Sample dilution** | **Observed (ng/ml)** | **Expected (ng/ml)** | **Recovery (%)** |
| --- | --- | --- | --- |
| 1:2.5 | 20.48 | 12.50 | 169.40 |
| 1:10 | 12.87 | 12.50 | 106.45 |
| 1:50 | 11.81 | 12.50 | 97.69 |
| 1:100 | 13.59 | 12.50 | 112.41 |


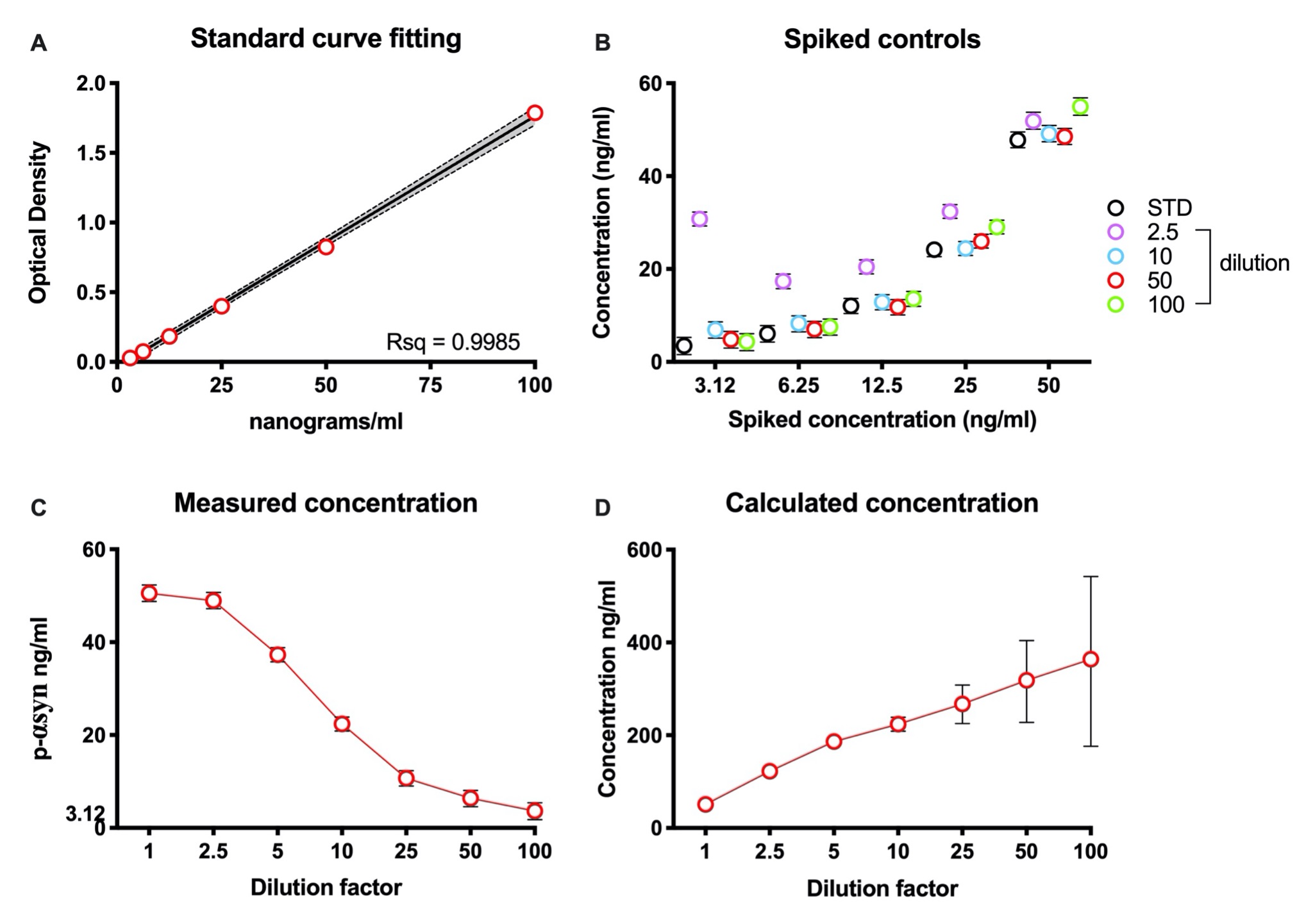


**Figure S1:** Pooled samples were used to set up the ELISA assay for the detection and quantification of the phosphorylated form of αsyn (p-αsyn) in saliva. (A) The standard curve based on known concentrations was used to calculate the concentration in each sample; (B) several concentrations of standard (recombinant human p-αsyn) were spiked into samples in order to assess the assay suitability for quantifying p-αsyn in saliva. (C) Raw measured concentrations were corrected by multiplying for the dilution factor (D). Interpolated data are reported as mean

**Correlation analysis**

The Spearman's Rank correlation coefficient was used to check for correlations between clinical data and the concentrations of αsyn forms and ratios. We found that tot-αsyn was negatively correlated with UPDRS-III (r=-0.7785, p=0.0025; Fig S2). In addition, the p-αsyn showed a negative correlation with NMSS (r=-0.6154, p=0.0165; Fig S2). Finally, any correlations was found between the o-αsyn or αsyn ratios and the PD patients’ clinical data.

**Figure S2:** Statistically significant correlations between salivary concentrations of tot-αsyn and UPDRS-III, and between p-αsyn and NMSS, respectively. The data were analysed by Spearman's Rank correlation coefficient and were considered significant when p<0.05.
